# Abscess of the Female Pelvic Organs*Read at meeting Brazos Valley Medical Association, Cameron, Texas May 9 and 10, 1905.

**Published:** 1905-10

**Authors:** J. P. Oliver

**Affiliations:** Caldwell, Texas


For Texas Medical Journal.

### Abscess of the Female Pelvic Organs.*

BY J. P. OLIVER, M. D., CALDWELL, TEXAS.

It has been said, and perhaps correctly, that the study of the female cadaver has been the source of more scientific information on the pathology and therapeutics of the female system than any other. While this may be true in the abstract or last analysis of the diseases of the female pelvic cavity, it only reveals secondary pathological changes and not primary. However, there is ample evidence to prove beyond contradiction that the cadaver investigations of the female pelvic organs have resulted not only in a better understanding of the causes of these diseases, but a vast improvement in treatment and cure.

Not to say anything about the relief of suffering women from the pain incidental to child-birth and other causes, the subject under consideration is one of supreme importance to every member of the medical profession, whether a specialist or a general practitioner. Not unfrequently women carry pus tubes, suppurating ovary cysts and encapulated sacs of pus for months and some for years before correct diagnoses have been made. The space between the uterus, bladder and vagina above, and the vagina and rectum beneath, as well as the broad, round and sacro-uterine ligaments is filled with connective or areola tissue which serves physiologically to steady and break the shock, so to speak, which is imparted to the pelvic organs in walking and other causes.

---

*Read at meeting Brazos Valley Medical Association, Cameron, Texas May 9 and 10, 1905.

Mr. Duncan in his work on the diseases of women, says that perimetritis and parametritis is the stock in trade of the gynecologist. These two diseases have been, and are still, the terror of the puerperal and non-puerperal states; have caused many women to become sterile, many removals of the uterine appendages and untold numbers of cases of social and domestic unhappiness. There is said to be three forms of cellulitis,—exudative, phlegmonous, and suppurative. The first results in serous effusion; the second in adhesions and displacement of the uterus and its appendages, and the third in suppuration. The second variety is a hard, board-like condition of the roof of the pelvis and the surrounding parts, is very common, the results of which ultimately almost disappear by absorption, the remnants of which sometimes are mistaken for small fibroids upon the exterior of the uterus at different points. In the third or suppurative form, the tendency of the pus is to burrow and seek an outlet at the most vulnerable point, or that of least resistance, the most usual route being into some one of the hollow organs,—the bladder, vagina or rectum,—vagina first, rectum next, or it may extend upward and seek an outlet at the inguinal canal, especially if complicated with appendicial trouble. Cellulitis usually occurs on either side of the uterus in the connective or areola tissue, in the folds of the broad ligament, from ten days to two weeks after suppuration. It may occur much earlier, owing to the septic or aseptic condition of the parts, and usually supervenes upon a local traumatism of some portion of the genital tract, or an imperfect removal of the debris of abortion or parturition and a sepsis is admitted through lymph channels, producing perimetritis or parametritis of the endo- or parametrium; the left side is said to be involved more frequently than the right. The entire pelvic areola or connective tissue may become involved; this is not the rule, however, but the exception, Why the left side should be the most vulnerable point of attack, I am unable to say, unless it be because there is no valve in the left side at the junction of the spermatic with the left renal vein. This anatomical condition, it is reasonable to conclude, permits or favors blood stasis by gravitation into the radicals or venules of the spermatic vein, or there may be an easier transmission of micro-organisms through the blood and lymph channels into the endo- and parametriums, and the result being infection by pus-bearing germs.

Pelvic abscess is sometimes remote; that is, it involves the submucous coat of the peritoneum, but maintains its connection with the uterus, pushes it down to the brim of the pelvis, as existed in one of the cases reported by Matthews Duncan, the patient being an invalid of seven months. It would seem that any general practitioner or surgeon in the dawn of the twentieth century who would permit his patient to carry a pus sac or a suppurating hematocele for seven months would subject himself to severe criticisms if not prosecution. Yet, Mr. Duncan's footprints in St. Bartholomew's Hospital will be long remembered. The point most desired in this connection is to impress, if possible, great care in the diagnoses of all possible or probable cases of pus formation in the pelvic cavity of the female.

Ischio-rectal abscess in the male is frequently found in connection with fistula, either blind or open. This is only referred to incidentally, and does not come specially within the scope of this paper. The fluid elements of encysted pus formation of the female pelvic organs is sometimes absorbed, the residue being caseated, is not infrequently mistaken for small fibroids, especially if the glandular structure is involved. Dr. Duncan further states, of all the prolific causes of parametritis of which he has any knowledge, inflammation of the mucous membrane of the uterus is the most common, and this in both the puerperal and non-puerperal state. Why should this be so? Because both are subject to acute and chronic cellulitis. It must be remembered that Drs. Duncan, Hewitt, Thomas, Emmett, and Byford and many other gynecologists wrote anterior to the micro-organism knowledge of infection, hence the discrepancy, or apparent discrepancy, in the pathological conditions mentioned by these gentlemen and gynecologists of the present day. Mr. Emmett says very great advances have been made of late years in uterine pathology and therapeutics. I maintain that a close study of the metrium and parametrium and the results will keep the profession better informed about pelvic troubles in the female and móre satisfactory results will be obtained from treatment.

Inflammation of the endometrium and parametrium is, perhaps, the most frequent cause of pelvic abscess of the genital tract in the female. These conditions, as above stated, may follow abortion, especially criminal, parturition, hematoceles, ovarian cysts or traumatism of any portion of the genital tract. Acute cellulitis may or may not be ushered in with a chill, the temperature rises from one to five degrees, pain is often referred to the lower abdomen or to either side; temperature in the vagina is generally one degree higher than the armpit. If the attack be on either side, pain is referred to that side; if general, all over the lower abdomen and painful to the touch, and usually considerably tympanitic. Many other symptoms are frequently present, and the judgment of the attending physician is taxed not infrequently to the utmost degree, because he recognizes the gravity of the case and the impending results. If the patient is seen early and a correct diagnosis is made before pus is formed, the bowels should be opened with a saline or mercurial purge. The latter, perhaps, is the best in most of the cases. Should diarrhea or pain of a persitent character be present they will demand prompt attention. The uterus should be cleaned of all debris and then irrigated two or three times daily with a warm, sterilized solution of either lysol, 1 to 3000, or bichloride of mercury, 1 to 4000, and an antiseptic pad of absorbent cotton and iodiform gauze applied fresh after each irrigation. After using the germicidal wash, warm, sterilized water should be used for warm normal salt solution. Each of the antiseptic solutions have their advocates, but I prefer the bichloride. If the case is seen early and irrigation followed as above directed, especially if administered by a trained nurse or the physician himself, oftentimes you will be surprised to see how soon the temperature will begin to decline; especially is this so if strict asepsis is maintained throughout the treatment of the case. If the case terminates in serum or sero-pus formation, a large majority of the authors to whom I have referred, advise the use of an exploratorial needle to confirm diagnosis and then insert sharp-pointed scissors beside the needle and discharge the abscess through the vagina to more radical measures.

Maintain strict asepsis, irrigate the abscess cavity as above directed, and the results usually will be satisfactory to all parties. This accords with my experience in a case treated successfully which I shall report below. Mr. Tate, than whom I know of no better authority on abdominal surgery in females, reports six cases of suppurating hematocedes, all successfully treated by abdominal section, but he styles it his new plan, and says that until recently he has confined his treatment to opening through the vagina in the neighborhood of Poupart's ligament. Experience, he says, has driven him to the conclusion of Dr. Emmett, "that I can not regard the introduction of the trocar into the inflamed pelvic tissues free from danger under all circumstances." Howard Kelly, a gynecologist said to be Mr. Tate's equal, if not his superior in abdominal work, says the causes of suppurative affections of the pelvic organs of the female are due to any of the pus-producing micro-organisms, which usually find entrance through the vagina into the uterus, thence into the pelvis by way of the uterine tubes, or the lymphatics into the paremetrium. Finally Mr. Kelly advises the vaginal route of discharging the abscess pus to more radical measures. Of sixty-five cases reported in his work, there were fifty-five cases of pelvic abscess, ten of clearly defined pyosalpinx; of the sixty-five cases opened through the vagina, thirty-two were cured, two died, some very much relieved, and a small portion no better. Hence, preponderating evidence favors the vaginal route of discharging the pus from a pelvic abscess in the female from whatever cause to more radical measures, much easier performed, not so grave to the patient, and I believe gives better results. Some cases whose environments are different, demand more radical measures; the case submitted below was so much emaciated I am sure abdominal lesion would have resulted in the fatality of my patient, who now enjoys fairly good health.

July 27, 1904, was called to see Mrs. G., colored, about 45 years of age and married; mother of four children; eight years since the birth of the last. The trouble for which I was called was malarial fever and irregular menstrual flow, which, according to her statement, had existed for some time. There was no history of any specific disease or constitutional vice. Digital examination revealed a patulous os with some erosions. The uterus was curetted, local application of iodine and carbolic acid made to the endometrium. During the examination a round tumor about the size of a small orange was detected in the region of the left ovary. I told her that it might require an operation for its removal, as there was no fluctuation that could be detected. Hot vaginal carbolized douches was advised daily. This woman was some days better, some worse; fever continued all the time, running from 104 to 99, never, perhaps, clear of fever. The flow from the uterus still kept up in spite of everything that was done. Dr. Scott, of Temple, saw this patient one time with me, and remarked that it was not only very peculiar, but very interesting, also; made no suggestions further than what I was doing. On the 28th of August the uterus was again curetted with a dull or blunt curette. On the 4th of September, to my surprise, my patient aborted a foetus of about two months. The uterus was examined, and seemed to have been well cleared, as everything together with large clots of blood was in the chamber. Strict asepsis was observed, and for a few days my patient seemed somewhat relieved. The tumor, however, still remained. September 9 vaginal examination revealed a hard, round mass pressing against the posterior vaginal walls, seemingly perfectly hard, and no fluctuation could be detected. A hypodermic needle was introduced to ascertain whether or not the tumor contained pus, which was verified on the withdrawal of the needle. A narrow-pointed bistoury was introduced and the pus discharged through the vagina. On the 12th, Dr. Kruger assisted me, and the vaginal opening was dilated with a large uterine dilator, and gauze drainage inserted. The next day the gauze was removed and a three-eighths rubber tube was inserted, which drained the abscess cavity well. The abscess cavity was irrigated daily with a bichloride solution, 1 to 4000, followed by warm sterilized water eight or ten days, and the patient discharged.

